# Deep learning-assisted concentration gradient generation for the study of 3D cell cultures in hydrogel beads of varying stiffness

**DOI:** 10.3389/fbioe.2024.1364553

**Published:** 2024-04-11

**Authors:** Vasileios Anagnostidis, Anuj Tiwari, Fabrice Gielen

**Affiliations:** ^1^ Living Systems Institute, Faculty of Health and Life Sciences, University of Exeter, Exeter, United Kingdom; ^2^ Department of Physics and Astronomy, Faculty of Environment, Science and Economy, University of Exeter, Exeter, United Kingdom

**Keywords:** microfluidics, deep learning, concentration gradient, 3D cell culture, object detection, stiffness, hydrogel

## Abstract

The study of dose-response relationships underpins analytical biosciences. Droplet microfluidics platforms can automate the generation of microreactors encapsulating varying concentrations of an assay component, providing datasets across a large chemical space in a single experiment. A classical method consists in varying the flow rate of multiple solutions co-flowing into a single microchannel (producing different volume fractions) before encapsulating the contents into water-in-oil droplets. This process can be automated through controlling the pumping elements but lacks the ability to adapt to unpredictable experimental scenarios, often requiring constant human supervision. In this paper, we introduce an image-based, closed-loop control system for assessing and adjusting volume fractions, thereby generating unsupervised, uniform concentration gradients. We trained a shallow convolutional neural network to assess the position of the laminar flow interface between two co-flowing fluids and used this model to adjust flow rates in real-time. We apply the method to generate alginate microbeads in which HEK293FT cells could grow in three dimensions. The stiffnesses ranged from 50 Pa to close to 1 kPa in Young modulus and were encoded with a fluorescent marker. We trained deep learning models based on the YOLOv4 object detector to efficiently detect both microbeads and multicellular spheroids from high-content screening images. This allowed us to map relationships between hydrogel stiffness and multicellular spheroid growth.

## Introduction

Quantifying the relationships between the concentration of a molecule and its effects on a target biological system is crucial for advancing the field of quantitative biology and building predictive models for the response of living systems to effectors (Howard, 2014; Booij et al., 2019). Advances in miniaturization technology, such as microfluidics, make it possible to generate parallelized, combinatorial and high-throughput assays (Cao et al., 2012; Gielen et al., 2013; Neun et al., 2022). Droplet microfluidics, in particular, provides the capability to produce and test myriads of experiments simultaneously with stable micro-environments in which encapsulated living cells can proliferate.

An important application of droplet microfluidics is the production of hydrogel beads in which cells are encapsulated (Allazetta and Lutolf, 2015; Kleine-Brüggeney et al., 2019). The gels provide porous, inert scaffolds, partly mimicking the mechanical properties of the extracellular matrix (ECM) (Madl et al., 2018). Hydrogels can be prepared with biocompatible polysaccharides (natural or synthetic) with various modes of gelation (e.g., induced by changes in pH, temperature, presence of chelators…) and can be dissolved in aqueous phases in different concentrations to produce gels of varying stiffness (Yang et al., 2022).

The relevance of the mechanical properties of cellular micro-environment to cell phenotypes and drug response is already well established (Hayaei Tehrani et al., 2021). Mechanosensing is recognized to play an important role in cellular and embryo development with many signalling pathways involving stiffness sensing (Martino et al., 2018). For example, ECM stiffness affects cancer cell proliferation and tumour drug response (Cavo et al., 2016). Studying the relationships between cellular growth, self-organization, and homeostasis in niches of varying stiffness is therefore crucial to uncover the contribution of mechanical cues to emerging phenotypes. This knowledge will ultimately help design organoid models that can more faithfully mimic human physiology (Li and Kumacheva, 2018a).

To date, there is a lack of methods to systematically investigate stiffness-growth relationships beyond low-throughput techniques. Droplet microfluidics holds the potential to provide large scale assays but altering drop-by-drop composition with high control remains technically challenging. Current methods require complex engineering such as the use of droplet-on-demand platforms, making use of Taylor-Aris dispersion combined with droplet formation, or the coupling of droplets with other analytical instruments that separate chemical mixtures (Du et al., 2010; Theberge et al., 2010; Miller et al., 2012; Gielen et al., 2015). Conventional droplet microfluidic devices can be also used for the rapid encapsulation of multiple assay components, by co-flowing solutions before droplet formation, albeit with lower degree of control and dynamic range than counterpart systems (Song and Ismagilov, 2003; Hess et al., 2015). In such implementation, continuous variation of the pressure or flow rate provided by pumping elements enable continuous change of the stoichiometry of multiple co-flowing reagents before encapsulation into droplets where efficient mixing occurs. A practical limitation arises when pumping fluids of high viscosity (i.e., orders of magnitude more viscous than water) (Gielen et al., 2013; Hamidovic et al., 2020; Wong et al., 2020). Indeed, high resistance to flow within microchannels and compliance of the fluidic system (e.g., deformable devices, soft tubings) lead to hard-to-predict output flows coupled to potentially unstable droplet formation regime (Perry et al., 2010; Moreira et al., 2021). For hydrogels such as sodium alginate, apparent viscosity for a range of grades dissolved at 2% w/w in water was found to be an average of 900 cP compared to 0.89 cP for water only at 25 C (Fu et al., 2011). This issue can be alleviated by designing real-time flow monitoring systems able to quantify and adjust flows.

Recent advancements in machine vision technology, facilitated by the use of high-performance graphics cards and efficient image analysis algorithms, have paved the way for the development of self-correcting and operation-on-demand microfluidic systems (Crawford et al., 2017; Anagnostidis et al., 2020a; Howell et al., 2021; Luo et al., 2022; Tiwari et al., 2023). These systems utilize real-time imaging feedback to monitor the progress of an experiment and adjust the state of actuators to achieve a desired output. This development is anticipated to provide rapid and intelligent feedback on microfluidic function towards fully unsupervised systems but to date has not been applied to producing concentration gradient coupled to droplet formation (Srikanth et al., 2021).

Here, we demonstrate how a conventional microfluidic co-flow device can be coupled to a closed-loop deep learning-assisted feedback system to generate controllable hydrogel concentration gradients. We used a shallow convolutional neural network model to evaluate the position of boundaries between two laminar flows. This allowed us to generate and screen thousands of HEK293FT 3D cell cultures growing in alginate beads of stiffness varying between 50 Pa to close to 1 kPa encoded by a fluorescent reporter. We quantified cell culture growth over 8 days using high-content imaging. Image datasets were rapidly analyzed with deep learning object detector YOLOv4 to obtain quantitative relationships between stiffness and multicellular spheroid growth.

## Materials and methods

**Cell culture.** The human embryonic kidney (HEK) cell line 293FT was cultured in Dulbecco’s Modified Eagle Medium (DMEM, Gibco) supplemented with 10% Fetal Bovine Serum (FBS, Gibco) and 1% Glutamax (Gibco). The medium was filtered through a 0.2 μm filter (Sartorius) before usage. Cells were passed when reaching approximately 80% confluency using 0.05% trypsin-EDTA (1X) (Gibco) to detach the cells from the surface of a T25 flask before resuspension in fresh medium by centrifugation at 1,200 rpm for 5 min.

**FIGURE 1:**
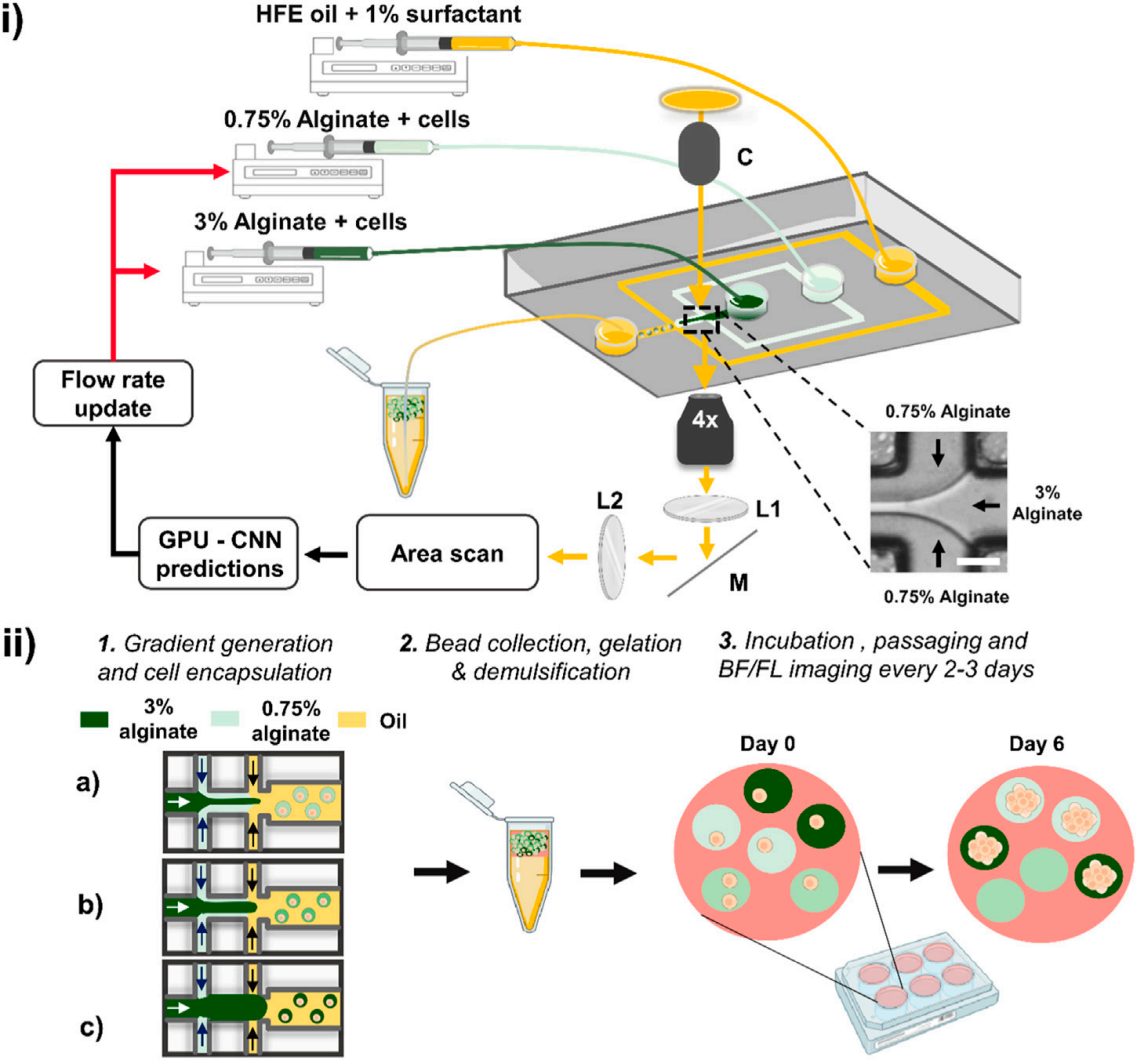
Schematic of the deep learning-assisted feedback method for the generation of alginate concentration gradients. **(i)** The method relies on a microfluidic device in which two aqueous phases co-flow followed by droplet formation at a flow-focusing junction. The junction at which the two aqueous solutions meet (inset) is imaged using an inverted microscope with transmitted light passing through a collimator (labelled ‘C’). The light is directed along the imaging path towards an area scan camera passing through two relaying lenses (L1 & L2) and a mirror (M). Plano-convex lenses L1 and L2 had a focal length of 50 mm. Individual frames were acquired by the camera and passed through a pre-trained CNN. The CNN evaluations were used to adjust the flow rates of the syringe pumps. Scale bar: 50 μm. **(ii)** Schematic of the workflow for cell growth in beads of varying stiffness. Different volume fractions are produced in sequence (as depicted in a, b and c), and the droplets were collected in a tube in which gelation occurred. The beads were then demulsified, suspended in culture medium and dispensed in a well plate placed in a cell culture incubator. The beads were regularly imaged in bright-field (BF) and fluorescence (FL) modes using a high-content screener to monitor spheroid growth across all alginate stiffnesses.

**Optical setup and electronics.** We used an inverted microscope (IX73, Olympus) and a fast area scan camera (Pike F032B) to acquire images. A white LED light source (CoolLED, pE-100) was collimated towards the microfluidic device and directed towards the camera via two relay lenses (both with focal distance of 50 mm). Exposure time and gain of the camera were set at 80 μs and 5 dB, respectively. Optical parts and connectors were purchased from Thorlabs. A field-programmable gate array (PCIe-7841, National Instruments) was used to provide a continuous 50 Hz trigger signal to the camera. An area of interest showing the laminar flow at the interface between the two aqueous solutions was selected (as shown in Figure 1i, inset) and images of size 120 × 120 pixels were collected at ×4 magnification. To train the convolutional neural networks (CNNs), we used a Windows 10, 64-bit operating system with an Intel i5-6500 3.2 GHz processor with 32 GB RAM and CUDA capable dedicated graphics card (GeForce 1080 GTX Zotac). Images and classification results were saved in real-time on a local SSD drive as previously reported (Anagnostidis et al., 2020b). For the gradient generation, we combined the CNN model and syringe pump commands (Nemesys, Cetoni) in Python 3.7 using TensorFlow 2.3.0 and OpenCV 4.1.1.26 libraries.

**Microfluidic device and preparation of alginate solutions.** A flow-focusing device (width: 80 μm, height: 80 μm) was fabricated using soft lithography techniques to produce monodisperse droplets (Duffy et al., 1998). The device comprised of an inlet for the continuous oil phase, two inlets for aqueous mixtures and a collection outlet (Supplementary Figure S1). To achieve a gradient of alginate concentration, two alginate solutions were prepared. A low concentration alginate solution was prepared consisting of 0.75% (w/v) alginate (Merck, medium viscosity alginate, W201502) directly dissolved in a solution of 200 mM CaCl_2_, 200 mM ethylenediaminetetraacetic acid (EDTA) and 100 mM 3-(*N*-morpholino) propane sulfonic acid (MOPS) in phosphate buffer saline (PBS) adjusted at pH 8.5. All chemicals were purchased from Merck. A final 1% (v/v) (125 nM) fluorescein isothiocyanate (FITC)-dextran (2 MDa) dissolved in PBS was added to the mixture and used as a fluorescence reporter encoding alginate bead concentration. The second solution consisted of 3% (w/v) alginate directly dissolved in 200 mM Zn^2+^, 200 mM thylenediamine-*N*,*N*′-diacetic acid (EDDA) and 100 mM MOPS in PBS at pH 8.5. A 5% (v/v) (625 nM) FITC-dextran dissolved in PBS was supplemented to the mixture. For the hybrid concentration gradient, we used 1% (w/v) agarose (Merck, A5030). Both solutions were supplemented with 10% (v/v) of HEK cells in culture medium to a final density of ∼6 × 10^6^ cells/mL. The continuous oil phase was prepared using HFE-7500 fluorinated oil (Fluorochem) containing 1% (w/v) 008-Fluorosurfactant (RAN Biotechnologies). Following alginate gradient generation, the generated emulsion was incubated at room temperature for 5 min and supplemented with 200 µL culture medium. The incubation period allows for ion exchange to take place, facilitating efficient crosslinking of the alginate into gel beads. The emulsion was subsequently demulsified using an antistatic gun (Zang et al., 2013). Beads were resuspended in culture medium and incubated in 24 well plates (Greiner) at 37°C with 5% CO_2_. The media was exchanged every 2–3 days after mild centrifugation at 300 rpm for 5 min to prevent damaging beads and spheroid escape. Images were taken using a high-content screener (HCS) system at 4x and 10x magnification (ImageXpress Pico, Molecular Devices) allowing for automated scans of entire wells (Supplementary Figure S2). The HCS was equipped with a precision motorized Z-stage focus hardware and software system. For bead imaging, an autofocus function was selected that searches the bottom of the well plate. Once the optimum focus was found, a further offset was manually adjusted for imaging the beads on both brightfield and fluorescence channels. A total of 5 wells were imaged corresponding to approximately 240 and 1,200 images per experiment at 4x and 10x, respectively.

**FIGURE 2:**
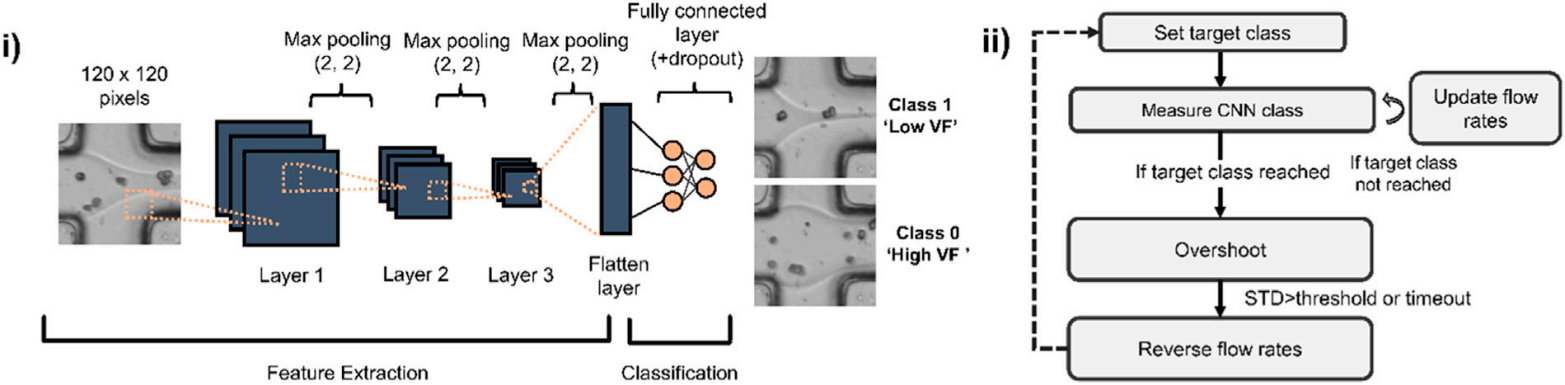
CNN architecture used for assessing microfluidic flows and state machine for gradient generation using CNN predictions. **(i)** A shallow CNN is trained to recognize whether a laminar flow interface is close to the centre (Low Volume Fraction (Low VF)) or edges of a microchannel (High VF). **(ii)** State machine used to automate the gradient making: a target class (either low or high volume fraction) is set and images of the laminar flow are taken at 50 Hz to estimate current CNN class and update the flow rates for both low and high alginate concentration solutions. When the target class is reached, an overshoot mechanism enables screening of flow ratios beyond those of the trained classes. When overshoot is completed, flow rates are reversed to progressively revert back towards the other class.

**Microfluidic gradient formation.** The process for alginate gradient formation started with using a pre-trained CNN model able to recognize low and high volume fractions (Figure 2i). We selected flow rates of 5 μL/min and 1 μL/min for the low and high concentration alginate solutions respectively (used to generate low volume fraction) and tested CNN predicted classes. In case of incorrect or low confidence classification, a new training set was collected and added to previous data for model retraining. This process, along with model training, lasted approximately 5 min. Once the model was satisfactory, flow rates of 3 μL/min and 2 μL/min were selected for the low and high concentration alginate solutions respectively, until a stable laminar flow was established. The continuous oil phase was run at a constant 30 μL/min. The droplets were generated at a rate of approximately 200 Hz for 5 min. Upper limits for flow rates (6 μL/min and 13 μL/min for high and low concentration alginate solutions respectively) were set to prevent long delays between successive gradients.

**Alginate bead and spheroid detection analysis.** We used the YOLOv4 object detector to localize both fluorescent beads and spheroids, with image processing taking 0.2 s per image. We assembled custom training datasets obtained from the high-content screener. Three separate models were created for beads and spheroid detection consisting of i) 10x fluorescence bead images, ii) 4x fluorescence bead images and iii) 10x bright-field spheroid images. A total of 85 images (with ∼6,200 beads), 11 images (with ∼1,500 beads) and 24 images (with ∼1,000 spheroids) were labelled from 10x data for beads, 4x data for beads, and 10x data for spheroids, respectively. Image datasets for training were pooled from different experiments to increase model versatility and detection accuracy. A stratified split was used to create the training set (70%) and test set (30%). YOLOv4 training was conducted using Python 3.7 on a cloud virtual machine provided by Google Colaboratory. The networks were then trained over a total of up to 5,000 iterations and the best weights were picked (Supplementary Figure S3). A custom MATLAB script was written to extract spheroid diameters from the YOLO bounding boxes and associate a fluorescence intensity value corresponding to the bead FITC-dextran content.

**Scaling from fluorescence to gel concentration.** We assumed a requirement of minimum 10% Ca^2+^ or Zn^2+^ content in the droplets to effectively cross-link alginate (corresponding to minimum concentrations of ∼20 mM). Beads with lower content in calcium or zinc were assumed not cross-linked. We extracted the lowest and highest fluorescence values from the beads, corresponding to the lowest (0.7%) and highest (2.8%) alginate concentrations. This allowed us to establish a linear calibration between fluorescence and alginate concentration for the rest of the beads.

**Rheological characterization.** The viscoelastic behaviour of alginate with concentrations ranging between 0.75% and 3% were assessed using a dynamic shear rheometer (Kinexus DSR + Rheometer, Malvern) with a parallel-plate geometry. The preload force was about 0.3 N, the shear strain was 1% and the oscillatory shear stress frequency was 1 Hz. For the alginate gelation, alginate (dissolved in 200 mM CaCl_2_, 200 mM EDTA, 100 mM MOPS in PBS, pH 8.5) was loaded on the bottom of the geometry stage. Gels were prepared with a volume ratio of 1: 1 Ca-EDTA to Zn-EDDA solutions and maintained constant for all measurements. Immediately after the solution mixing on-stage, a 20 mm diameter top plate was lowered to a final gap of 0.5 mm (parallel plate set up). The evolution of the shear moduli, *G*′ (storage, elastic component) and *G*″ (loss, viscous component), was recorded at 37 °C as a function of time for 5 min (10 s interval, n = 2) for every alginate condition.

## Results

We demonstrate the generation of deep learning-assisted concentration gradients using a classical flow-focussing device in which two hydrogels (final concentration of 0.75% and 3% w/w) mixed with HEK293FT cells co-flow before encapsulation into monodisperse droplets as shown in Figure 1i. We acquired real-time bright-field images of the laminar flow interface and trained a convolutional neural network for the evaluation of instantaneous volume fractions. Based on this feedback, the flow rates of the syringe pumps were updated, and this closed-loop control system led to the formation of controlled concentration gradients. We validated the platform by studying the growth of HEK293FT cells trapped into alginate microbeads (Figure 1ii) (Andersen et al., 2015). Following the formation of the alginate concentration gradient, corresponding to different gel stiffnesses, beads were subsequently cultured in 24-well plates and regularly imaged by a high-content screener to obtain a time-lapse evaluation of cellular growth across all beads. We targeted an average of more than 1 cell per droplet to maximize use of every bead formed.

The method we chose to generate alginate gel beads is the competitive ligand exchange crosslinking (CLEX) method which has been previously described (Håti et al., 2016). Briefly, calcium-EDTA/Zinc-EDDA complexes are mixed with alginate in which the calcium ions are released by intrinsic higher affinity of EDDA for binding calcium. This triggers the exchanging of ions allowing for the divalent calcium ions to bind to guluronate blocks of the alginate chains (Lee and Mooney, 2012; Cao et al., 2020), resulting in the gelation of the alginate. Using this method, we co-flowed a solution of alginate with Ca-EDTA (pH 8.5) with another one containing only Zn-EDDA (pH 8.5). The generated emulsion was incubated for 5 min, during which the divalent calcium ions coordinate guluronate assembly into tight aggregates. The relatively high pH of the solutions delays the gelation process by a few minutes, preventing clogging of the microfluidic chip.

**Fluorescence encoding.** Long-term cell growth experiments in varying stiffness require the ability to accurately quantify the concentration of alginate in a per-drop basis. In our experiments, this is hampered by the loss of spatial arrangement when collecting beads into off-chip containers. To overcome this issue, we used a fluorescent dye (FITC) attached to a high molecular weight molecule (dextran, 2 MDa) acting as concentration encoder stably trapped in the alginate matrix during the whole duration of the experiments. We used a low (125 nM) and high (625 nM) FITC-dextran concentration added to the initial 0.75% and 3% alginate solutions respectively.

**Deep learning-assisted gradient formation**. To implement real-time feedback and dynamic adjustment of flow rates, we continuously imaged an area of 80 × 80 μm^2^ (corresponding to 120 × 120 pixels images) at the junction where the two aqueous solutions (containing 0.75% and 3% w/w alginate) meet as shown in Figure 1i (inset). An interface can be seen as a laminar separation between the co-flowing solutions due to their marked difference in refractive index. We trained a shallow CNN to classify images as belonging either to a high-volume fraction class where the solution containing 3% alginate makes up at least 80% of the total width of the channel and a low volume fraction class in which the same solution makes up less than 20% of the width (Figure 2i). Approximately 600 images were used for training, equally distributed across both training classes. The initial training sets were acquired by manually setting high (8 μL/min) and low (1 μL/min) flow rates for either the 0.75% or 3% alginate solutions, both containing cells at the same density (∼6 × 10^6^/mL).

Given the high reproducibility of the appearance of laminar flows and cells, we opted to train a CNN model made up of only 3 main convolutional layers (Figure 2i). Model training took less than 1 min using an Nvidia 1080Ti GPU. Following training, the model was used for all subsequent experiments and drift correction was rarely required. However, in such a case, approximately 600 additional images were acquired and added to the existing training datasets used to build a new, updated model.

To compare the presented CNN approach to more standard edge detection methods, we analysed the response of a Canny edge detector, one of the most widely used edge detection algorithms. The analysis confirms that laminar flow edges in sub-optimal lighting conditions or obscured by cells would pose a significant challenge to evaluate volume fraction (Supplementary Figure S4).

A custom-written software linked CNN predictions to syringe pump flow rates such that a regular alternation between both CNN classes, corresponding to the two different gel volume fractions was achieved. In brief, the software acquired images at a rate of 50 per second and computed the average CNN class for 50 images, resulting in the flow rates being updated once per second. Flow rates were increased or decreased in fixed steps of 1 μL/min (except for reducing high viscosity flow where we used a step of 3 μL/min) until a target class was reached with over 95% confidence (Figure 2ii, ‘if target class reached’). In order to maximize the dynamic range of the concentration gradient, we implemented an overshoot function whose goal was to continue increasing or decreasing the volume fraction beyond the set classes for a small amount of time (the smallest time between 10 s or when the standard deviation of the predicted class exceeded a threshold of 40%, meaning the appearance of the interface differed significantly from the training dataset). Gel bead monodispersity was ensured by keeping the disperse to continuous phase flow rate ratio smaller than 1:2 at all times.

**FIGURE 3:**
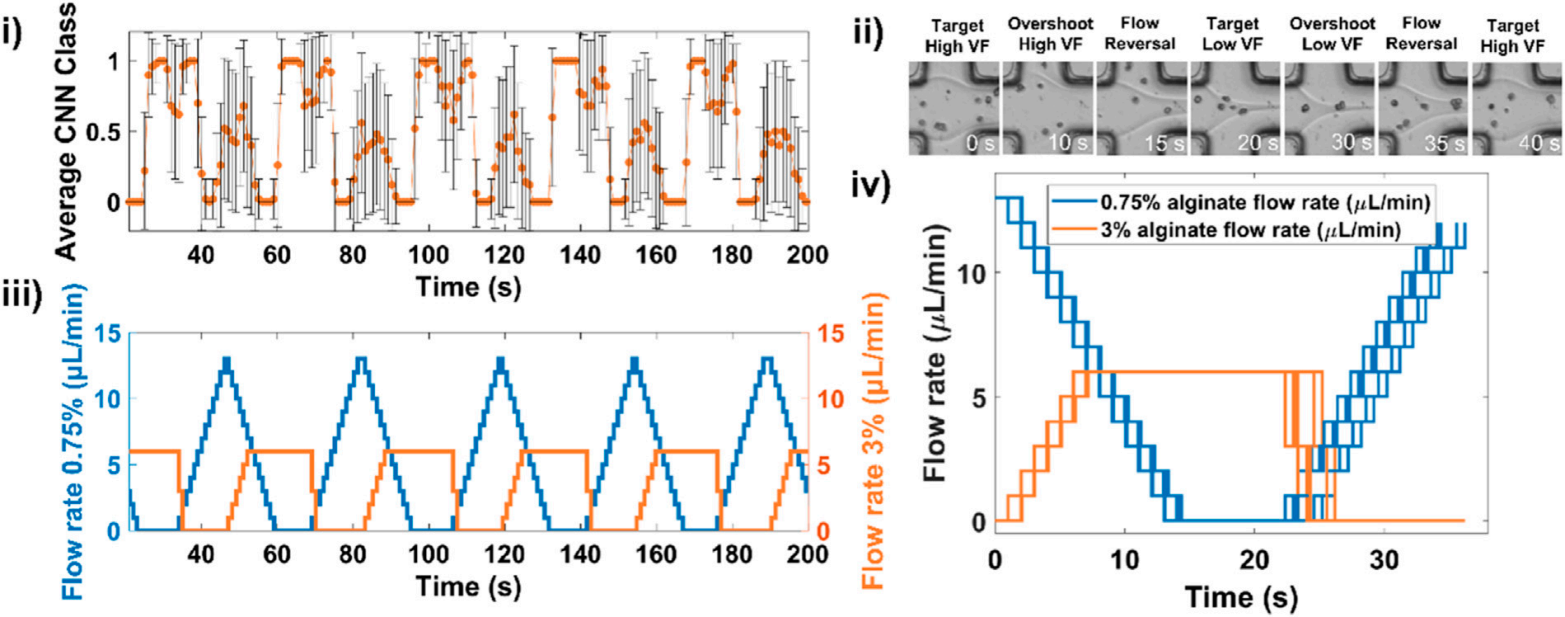
Deep learning-assisted microfluidic gradient generation during cell encapsulation. **(i)** An example of 5 consecutive gradients showing the measured CNN classes and **(ii)** corresponding evolution of flow rates for both low and high viscosity solutions. **(iii)** Example snapshots of the laminar flow junction at different stages of the gradient formation. **(iv)** Overlay of flow rate profile for 9 successive gradients.

An example of 5 consecutive gradients showing the alternation between low volume fraction and high volume fraction and the corresponding CNN-based feedback controlling syringe pump flow rates is displayed in Figure 3i,ii,iii and shows snapshots of the flow interface as classes alternate. The usefulness of CNNs in adapting to live experimental conditions is highlighted in Figure 3iv in which we plot flow rate profiles for 9 successive gradients. An example movie of a complete gradient is shown in Supplementary Movie S1.

### Long-term mechanical integrity of alginate beads

Alginate has been reported to gradually dissolve over time in various buffers including PBS (Alessandri et al., 2013). We tested possible dissolution (and therefore gradual decrease in stiffness over time) in cell culture medium by generating a control concentration gradient without cells. We passaged beads as in other experiments and imaged them with HCS using fixed imaging conditions (Supplementary Figure S5). The experiments revealed broadly stable FITC-dextran concentration during 7 days with a slight decrease after the first 24 h. Given we expected slight inconsistencies in focus and differences in background fluorescence for different days, we inferred gel concentration by assigning known low (0.7%) and high (2.8%) gel percentage corresponding to the 5th and 95th percentile of a Gaussian-fitted distribution of the fluorescence distribution histogram. We then applied a linear fit to relate fluorescence intensity to gel percentage for all beads.

### Assessment of bead stiffness

We assessed the viscoelastic properties of alginate at 37 °C by compression of thin hydrogel sheets using the same solutions as for the microfluidics experiments such that they can be used to provide accurate estimates of bead stiffnesses. The results show that the storage (Young) modulus increased from 50 Pa to close to 1 kPa for 0.7% and 2.8% alginate gels respectively (Supplementary Figure S6). The loss modulus was smaller than 5% the storage modulus, indicating a very small fraction of unpolymerized gel. The relationship between percentage of gel and storage modulus was not linear and we used a quadratic fit to derive gel stiffness from gel concentration.

### Spheroid formation in FITC-dextran labelled alginate beads

The average gel bead diameter was calculated to be 106 ± 24 μm. We chose an average density of around 3 cells per droplet (concentrating cells to a final density of 6 × 10^6^ cells/mL) to ensure maximum use of the gradient beads. Although we used a cell strainer when preparing cell solutions, some cell aggregates did form prior to encapsulation.

**FIGURE 4:**
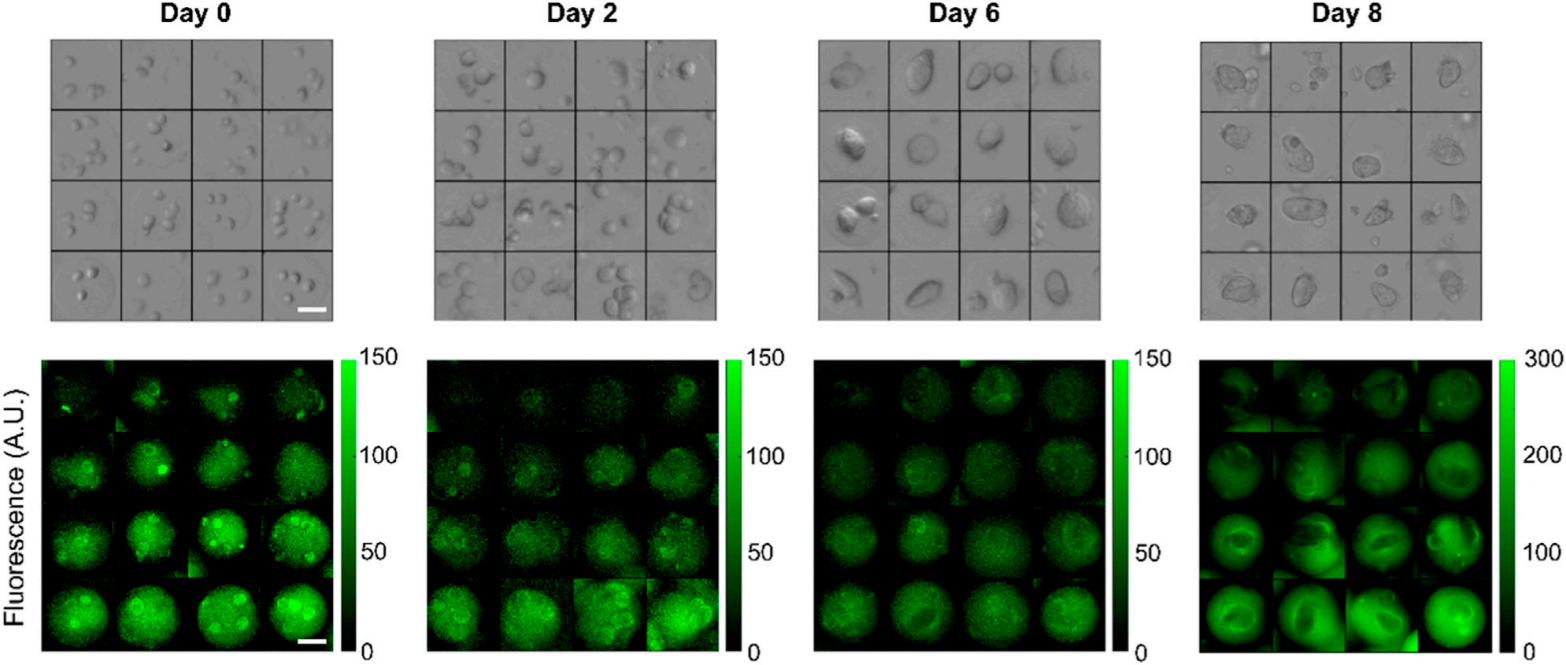
Representative bright-field and corresponding FITC-fluorescence images of individual beads containing HEK293FT cells forming multicellular spheroids over 8 days across different hydrogel stiffnesses. The stiffness gradient is encoded by a trapped fluorescent dye that stably stain the beads. In this example, imaging data for days 0–6 were acquired with a ×4 objective while day 8 was obtained with a ×10 objective. Scalebars: 50 µm.

In a typical experiment, 8 concentration gradients were generated in 5 min (∼35 s per gradient) with bead formation rate of about 200 Hz for a total of approximately 60,000 beads. All beads were incubated for 5 min at room temperature for gelation to proceed, demulsified and transferred into fresh culture medium, distributed equally into at least 5 wells of 24 well plates, and placed into a cell incubator (Figure 1ii). We acquired both fluorescent and bright-field images every 2–3 days over timescales of up to 8 days after which most multicellular spheroids were escaping the gels. In a typical experiment, we imaged several thousands of beads. Representative images of beads initially containing multiple single cells growing into spheroids are displayed in Figure 4.

### Evaluation of cellular growth in a range of scaffold stiffness

**FIGURE 5:**
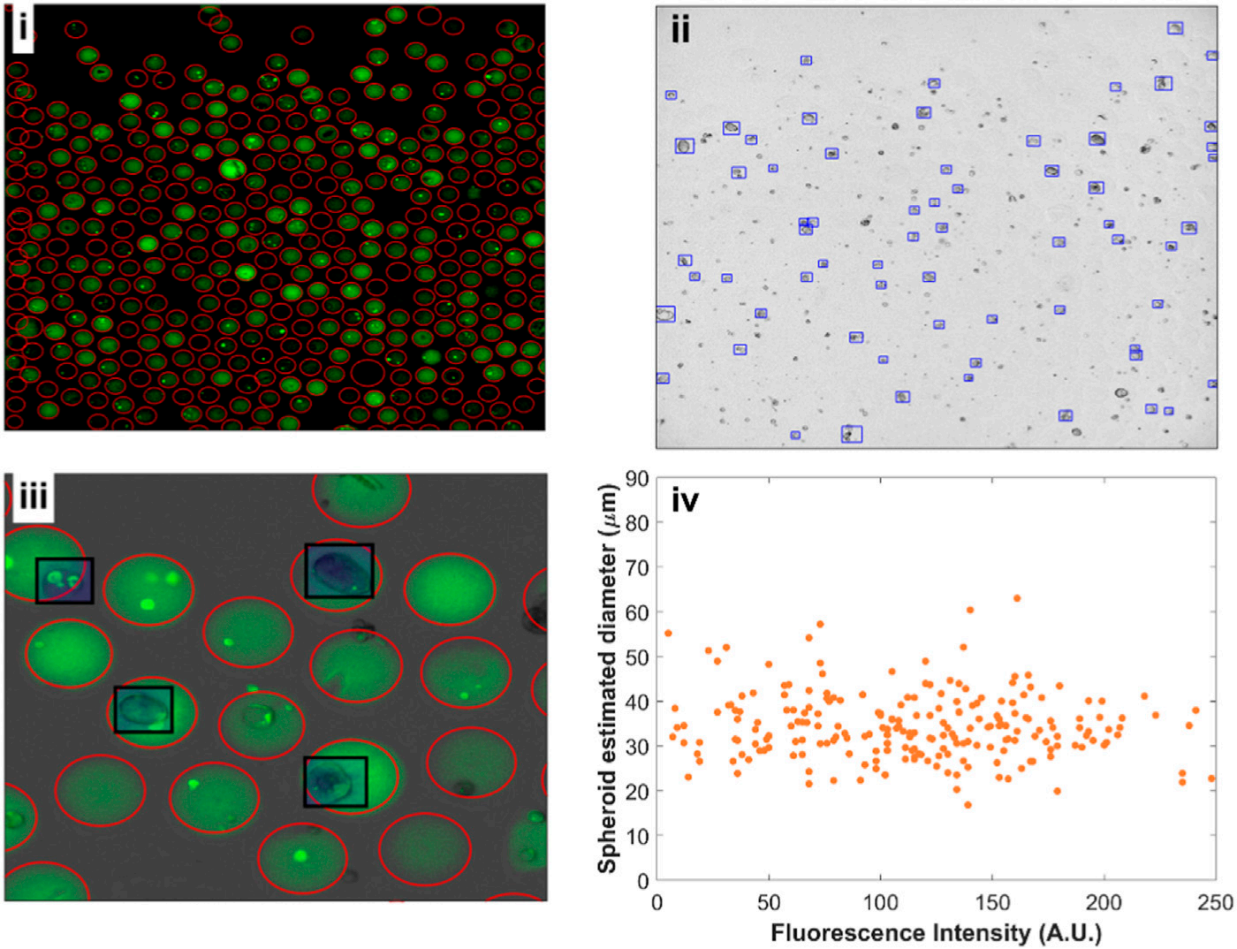
Fluorescent beads and spheroid detection using YOLOv4. **(i)** Fluorescence image (FITC) of alginate beads after 8 days of cell growth. 377 beads were detected in a single image. **(ii)** Corresponding bright-field image with HEK293FT spheroids grown in the different alginate concentrations. **(iii)** Close-up overlay of fluorescence and bright-field channels with beads (red circles) and spheroids (black rectangles) detections. **(iv)** Spheroid estimated diameter *versus* mean bead fluorescence intensity.

The encoding of the beads’ alginate content with fluorescent labels, as seen in Figure 5i, enabled us to assign gel concentration. However, many beads had a low fluorescence signal-to-background ratio, presumably because of partial leakage of the dye into culture medium and incomplete alginate cross-linking. This limited the accuracy of classical segmentation algorithms to detect such beads. To tackle this issue, we implemented an efficient deep learning object detector (YOLOv4) to identify fluorescent beads even with small signal-to-background ratio.

The distribution of the fluorescent dye within individual beads was found to be mostly uniform except, notwithstanding the presence of cells. We have further obtained confocal images of the beads at different alginate concentrations to confirm uniform 3D distribution of the dye following alginate cross-linking (Supplementary Figure S7). This enabled us to quantify the mean alginate concentration per bead with increased alginate concentration corresponding to higher stiffness.

We trained a separate deep learning YOLOv4 model to recognize spheroids from bright field images (Figure 5ii, iii). The bounding boxes given by the YOLO model for identifying spheroids allowed us to extract an approximate spheroid diameter. We assigned a fluorescence intensity corresponding to every detected spheroid, enabling us to plot estimated spheroid diameter against fluorescence intensity (Figure 5iv). Although we expect axial rotational symmetry and approximately spherical growth for the 3D cell cultures, obtaining high-resolution volumetric data would provide better quantification for spheroid growth, which can be done using confocal imaging. We have acquired example images of stained spheroids to exemplify possible volume measurements (Supplementary Figure S8). However, the long time necessary to obtain such images (∼1 min/image) precludes the rapid screening of thousands of beads.

**FIGURE 6:**
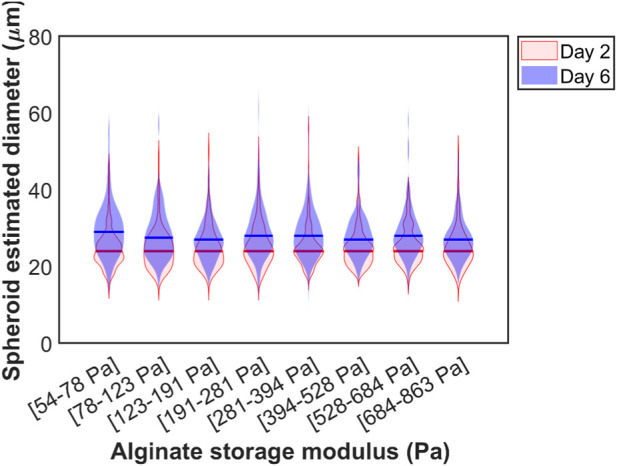
Violin plots for the spheroid estimated diameters against the stiffness of alginate at day 2 and day 6. The medians of the distributions are represented by horizontal lines of the corresponding color.

We repeated this analysis for the different days at which the images were acquired. To exclude spheroids with irregular shapes deviating from circular (e.g., when multiple spheroids fuse to form a larger one), we have only kept detections whose aspect ratio (length of detection box/width of detection box) ranges from 0.7 to 1.3. The comparison with and without filtering is displayed in Supplementary Figure S9. Figure 6 shows a violin plot for the filtered spheroid diameters across the estimated alginate stiffness gradient. We segmented the gradient into 8 separate bins for days 2 and 6. Each bin was made up of at least 65 spheroids. There were no statistically significant differences between medians of projected spheroid areas across the different alginate concentrations tested. A similar plot highlighting the growth of spheroids from day 2 to day 8 is shown in Supplementary Figure S10 with a larger number of spheroids exceeding diameters of 50 microns but similar medians for diameters across the gradient.

To further showcase the usefulness of the method for the generation of complex hydrogel systems, we also demonstrated the generation of hybrid agarose/alginate concentration gradients (Supplementary Figure S11). To do this, we incorporated an additional flow channel in the existing device to allow for another hydrogel to be added to the alginate phase. Specifically, we infused 1% agarose solution at decreasing flow rates for 5 alginate gradients in a row. The agarose solution was labelled with a TRITC-dextran dye, so that we could screen alginate/agarose combinations using dual-color imaging. We observed a non-uniformity in the distribution of both types of gels within individual beads creating complex topological landscapes. In this hybrid format, the assessment of local stiffness would be required to precisely map stiffness to cellular responses over time.

## Discussion

The proposed method for automated flow control in microfluidic devices based on imaging feedback is applicable, beyond hydrogels, to many sample pairs as long as the refractive index differ sufficiently to result in a visible laminar flow boundary.

The key advantage of using a CNN lies in its ability to provide automated and real-time feedback control without manual intervention. Traditional edge detection methods can be time-consuming and prone to errors, particularly in dynamic environments where conditions change rapidly due to the presence of cells, dust, air bubbles or pressure fluctuations. The CNN model was robust to the presence of dust or cells located directly on the laminar flow interface. Real-time adaptability is a challenge well addressed by CNNs as flow rates must be adjusted based on actual imaging feedback. Furthermore, the speed at which we analysed images (<20 ms per image) would be difficult to achieve with classical edge detection tools (Guo and Wu, 2023).

For mixing solutions made up with the same buffer, one could artificially increase the refractive index for one solution with an additive, e.g., sucrose (Zaca-Morán et al., 2018). The use of deep learning will be especially useful when dealing with low contrast interface between two solutions. In such cases, classical detection methods such as edge detection (e.g., Canny, Sobel…) would require extensive fine-tuning and the presence of cells in the gel phases would further complicate the detection of the flow interface.

In this work, we have trained a shallow CNN with 3 convolutional layers as fewer layers did not result in good models. Even though we chose to image the cross-junction where the two aqueous phases meet, one could train a model using images acquired at a different location of the chip, provided flow interface and volume fraction are still visible. The evaluation of CNN class at a rate of 50 Hz followed by averaging over 50 images was found to ensure accurate results and rapid adjustment of the flow rates once every second. In particular, this prevented sudden flow pulses to lead to premature class change, resulting in a more robust operation. Flow rate step increase and step decrease were chosen based on empirical observations but could also be adjusted based on the speed of transition between the two classes.

The average precision values achieved for the YOLOv4 models—based on 10x fluorescence bead images, 4x fluorescence bead images, and 10x bright-field spheroid images—were 99%, 88%, and 51% respectively (Supplementary Figure S2). While these scores indicate a high level of accuracy for fluorescence detections, it is worth noting that a significant proportion of spheroids were missed. This can be attributed to the fact that the fluorescent beads have very distinctive features (circularity, roughly uniform dye distribution). In contrast, the appearance (e.g., shape, size, texture) of spheroids in brightfield images showed higher variance and less distinct contrast, making their detection more challenging and leading to a comparatively lower average precision score. To address these limitations, augmenting the training dataset with additional images or tuning the network’s hyperparameters could improve detection accuracy. Yet, the obtained average precision scores were sufficient to localize the majority of spheroids and demonstrate the potential to establish accurate stiffness-growth relationships.

We showcase the use of FITC-labelled dextran polymers to encode alginate concentration and report on local gel stiffness.

The 2 MDa moiety is too large for direct cell permeation and escape from the gel matrix. However, when the plasma membrane becomes permeable, it can accumulate in the cells. For example, FITC-Dextran with molecular weight 4,000 Da has been used to identify apoptotic and necrotic cells by flow cytometry (Moumaris et al., 2015). This staining is visible in some single cells in Figure 4 indicating early apoptosis.

In this study, we image large wells in which beads sediment to increase data throughput at the cost of single-bead resolution. In this study, micro-cultures are pooled in standard wells of a 24 well plate. At adequate numbers (∼1,000–2000), beads sediment as a monolayer which can be conveniently imaged at the same focal plane for all cultures (Supplementary Figure S2). Adapting the method to single bead time-lapse imaging is possible but require the addition of a trapping and perfusion module that complicate implementation and reduce throughput (Kleine-Bruggeney et al., 2019; Tomasi et al., 2020).

**Decrease in the number of spheroids and beads over time.** We noted a steady decrease in bead number after each passage and attributed it to three factors: the incomplete pelleting of beads following centrifugation, the incomplete recovery from the wells and spheroid escape from the gel beads, presumably while being centrifuged. The rate of loss was found to be an equivalent average of 3%–5% per resuspension in fresh culture medium.

**Relationship between gel concentration and stiffness.** Several factors can affect the mechanical properties of the beads over time. Progressive degradation of the gels may occur although a study found no change in mechanical stability in standard culture medium until day 8 (Chui et al., 2019). In addition, stiffness may become anisotropic during spheroid growth, as cells push the gels outwards. Fluorescence anisotropies visible in Figure 4 were possibly triggered by cell growth.

Overall, cells experienced similar growth rates in the stiffness range tested. We assign the lack of statistical differences between spheroid growth to two factors: the chosen range of alginate stiffnesses below 1 kPa, considered ‘soft’ and the limited timescale for spheroid growth of 8 days. A study examining the growth of MCF-7 cells in alginate gels of similar stiffnesses found no significant growth differences for the first 8 days but observed differences from days 12–16 (Li et al., 2021). Although we were able to grow cells up to 12 days, we observed significant decrease in the number of spheroids due to escape from the gel beads. This could be solved in the future by generating larger hydrogel beads.

This is likely because the highest stiffness tested was too small to affect growth for this cell line (Aung et al., 2023; Bruns et al., 2023; Li and Kumacheva, 2018b). Our method will be therefore useful for probing soft tissues, e.g., mammary tissues with an elastic modulus between 160 and 200 Pa, adipose tissue (∼1 kPa), as well as bone marrow and lungs ECM (∼1 kPa) (Martino et al., 2018). Higher viscosity hydrogels or mixtures could be used to explore higher stiffnesses such as polyethylene glycol diacrylate, gelatin methacrylate, chitosan, gelatin (Baruffaldi et al., 2021).

The addition of natural or synthetic ECM components or native cell adhesion ligands have been reported for alginate microspheres (Lehnert and Sikorski, 2021). These methods provide more physiologically relevant models for studying gradient-dependent cellular responses in the context of organoids research. By incorporating biophysical cues and biochemical components, these developments are expected to pave the way towards the creation of hydrogels that better mimic *in vivo* cell environment.

## Conclusions and future work

Overall, the presented method demonstrates deep learning-assisted creation of on-demand, reproducible microfluidic concentration gradients without the need for empirical searches of optimum flow rate sequences and human supervision. The use of shallow CNNs for assisted gradient formation enables implementation of robust microfluidic functions and will be especially useful when using multiple high-viscosity solutions. The ability to obtain real-time feedback is crucial to ensure the success of complex microfluidic experiments operating over long timescales and will alleviate the need for constant supervision, eventually saving time and resources. Beyond the screening of relationships between matrix stiffness and 3D cell cultures, we foresee multiple applications of the method where establishing controlled dose-responses are essential such as forming gradient hydrogels, studying cell migration dynamics, or monitoring bacterial biofilm formation in gels.

## Data Availability

The raw data supporting the conclusions of this article will be made available by the authors, without undue reservation. https://github.com/GielenLab/Gradient_CNN.
