# Supplementary material for: Deep learning-assisted concentration gradient generation for the study of 3D cell cultures in hydrogel beads of varying stiffness
Source: Front Bioeng Biotechnol. 2024 Apr 11;12:1364553. doi: 10.3389/fbioe.2024.1364553 (PMC11044700; doi:10.3389/fbioe.2024.1364553)
Supplement: Supplementary file 1 [file DataSheet1.pdf]

Supplementary Information

Deep learning-assisted concentration gradient generation for the study of 3D cell cultures in hydrogel beads of varying stiffness

Vasileios Anagnostidis[1,2], Anuj Tiwari[1] and Fabrice Gielen[1,2,*]

[1] Living Systems Institute, Faculty of Health and Life Sciences, University of Exeter, Exeter, UK

[2] Department of Physics and Astronomy, Faculty of Environment, Science and Economy, University of Exeter, Exeter, UK

[*]Correspondence: f.gielen@exeter.ac.uk

Supplementary Figures

Figures S1-S11

Supplementary Movie

Movie S1

SUPPLEMENTARY FIGURES

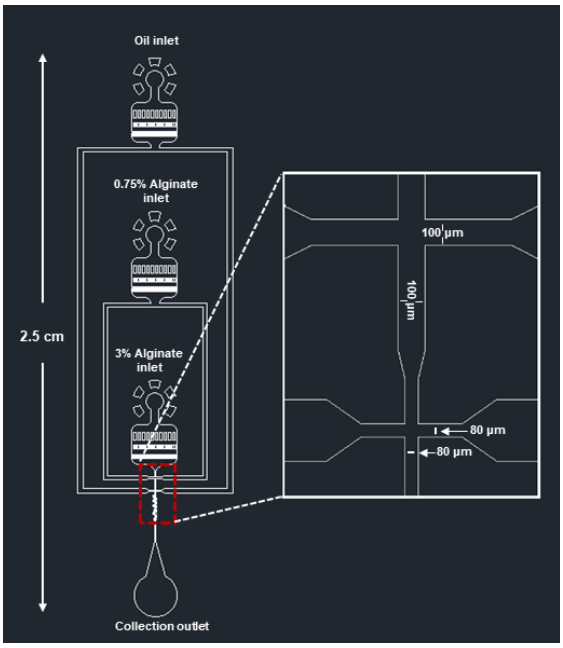

Supplementary Figure S1. CAD design of the flow-focusing gradient device with channel dimensions.

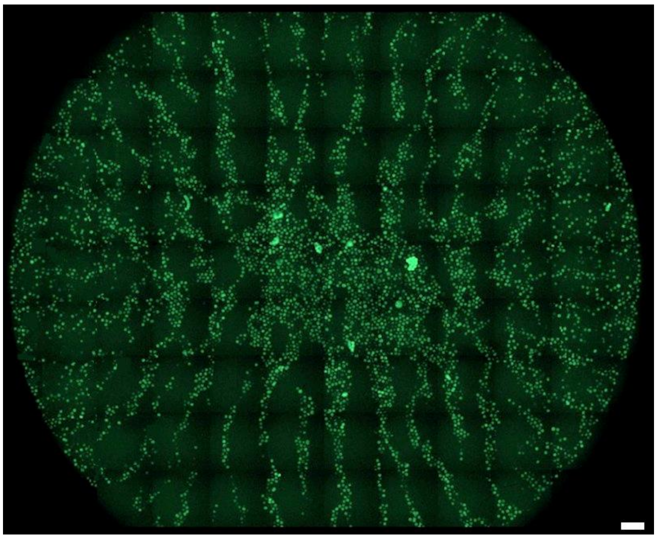

Supplementary Figure S2. Whole-well image acquisition of fluorescent alginate beads using a high-content screener (ImageXpress Pico, Molecular Devices) obtained by montage of 100 individual images acquired with a 10x objective. Over time, beads may slowly drift/accumulate at the center. Scale bar: 1 mm.

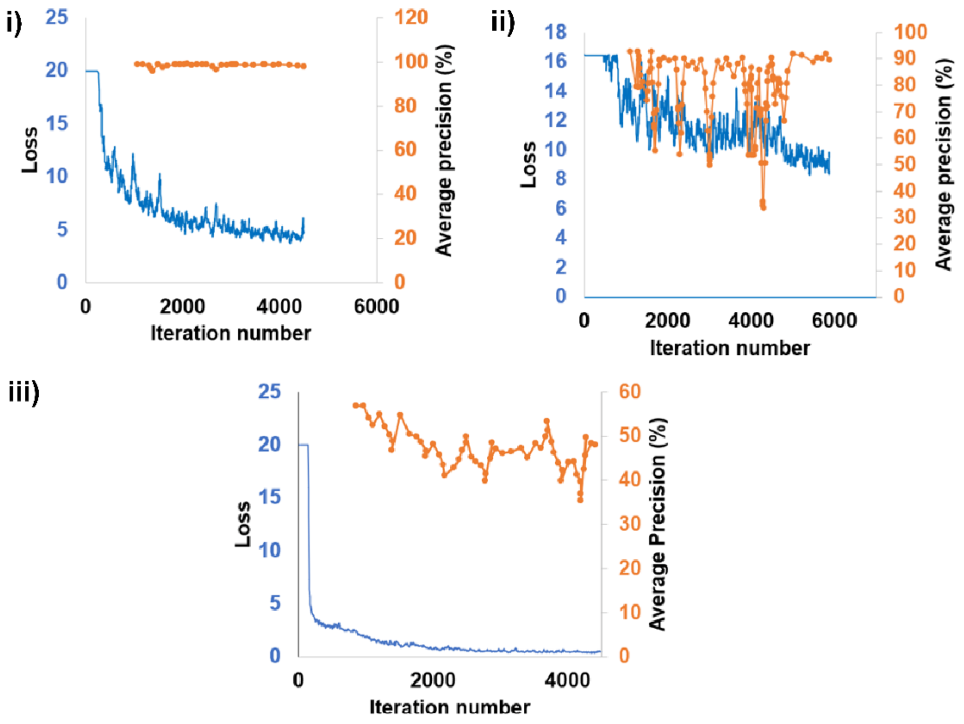

Supplementary Figure S3. Models' average precision as a function of training progress for fluorescent beads at 10x (**i**), 4x (**ii**) magnification and spheroids in bright-field images (**iii**). Weights used were the ones corresponding to the highest mAP and lowest loss.

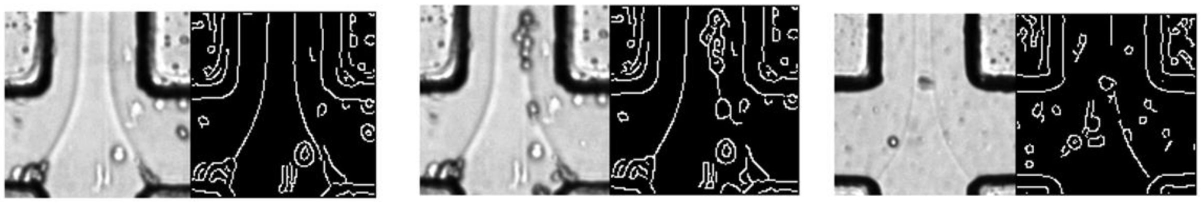

Supplementary Figure S4. Use of a Canny edge detector to evaluate laminar flow interface. **A.** Expected behaviour with both interfaces detected as a line separation. **B.** Cells present at the interface prevent identification of interface. **C.** Low contrast difference between the two aqueous phases result in absent or segmented flow edges.

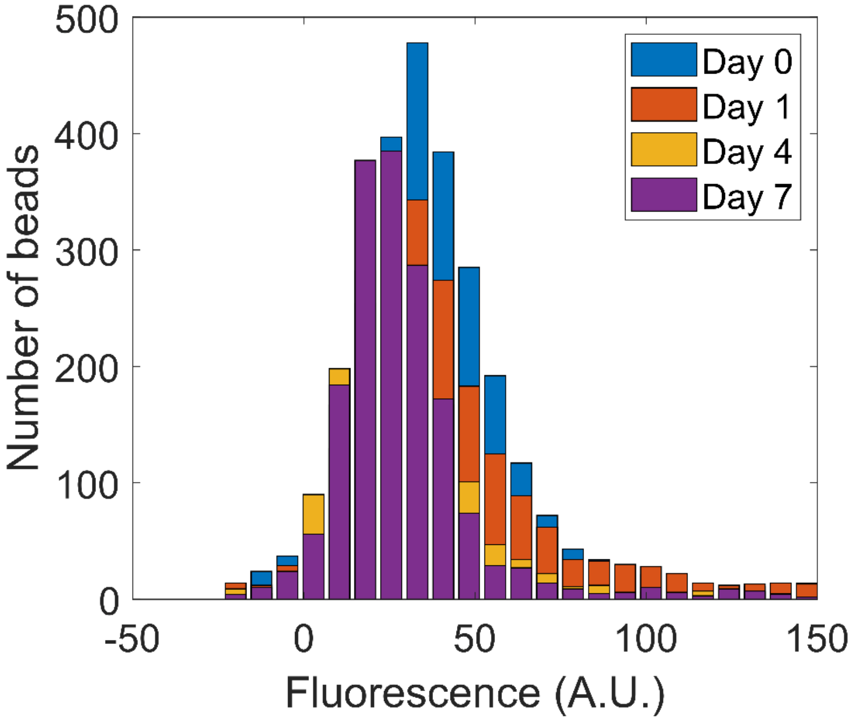

Supplementary Figure S5. Stability of fluorescence (FITC) distribution for 1.5% (w/v) alginate beads loaded with 2.5% v/v (312 nM) FITC-dextran labels and cells over 7 days.

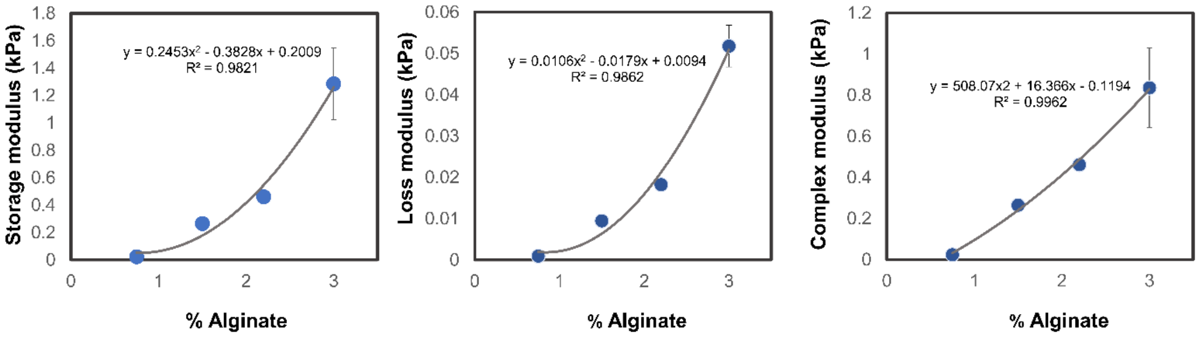

Supplementary Figure S6. Characterization of the mechanical properties of different alginate concentrations. They were assessed using a dynamic shear rheometer (Kinexus DSR+ Rheometer, Malvern) with a parallel-plate geometry. The preload force was about 0.3 N, the shear strain was 1% and the frequency (constant) was 1 Hz. Complex, loss and storage moduli measurements are displayed with N= 2.

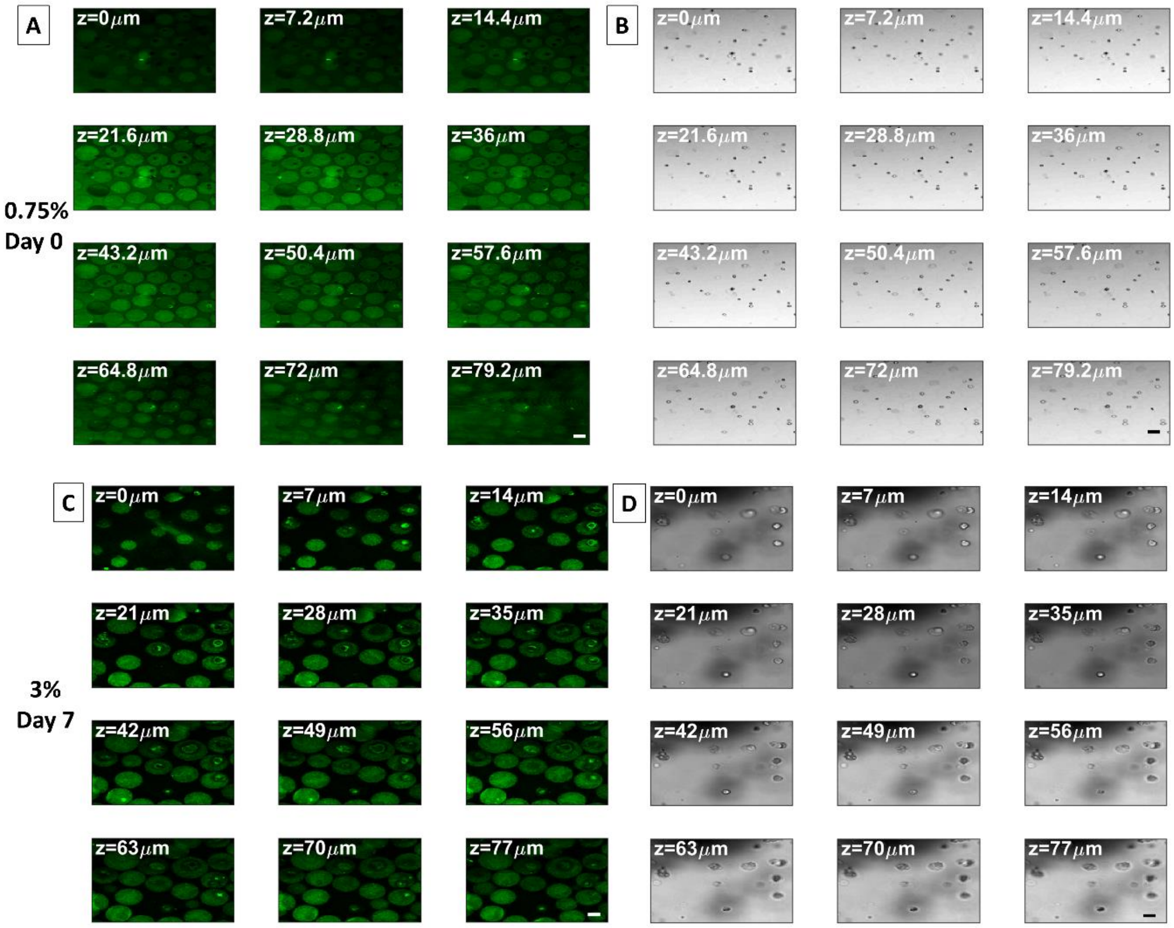

Supplementary Figure S7. Confocal images of HEK293FT cells encapsulated in alginate hydrogel beads. **A & B:** Fluorescence and bright-field pictures of 0.75% alginate beads at day 0. **C& D:** Fluorescence and bright-field pictures of 3% alginate beads at day 7. Scalebars: 100 µm.

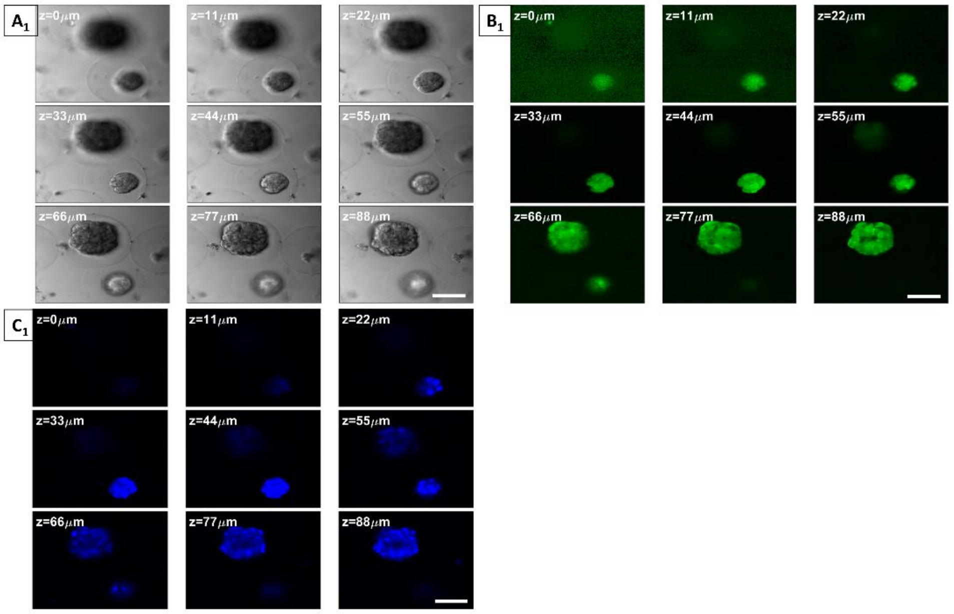

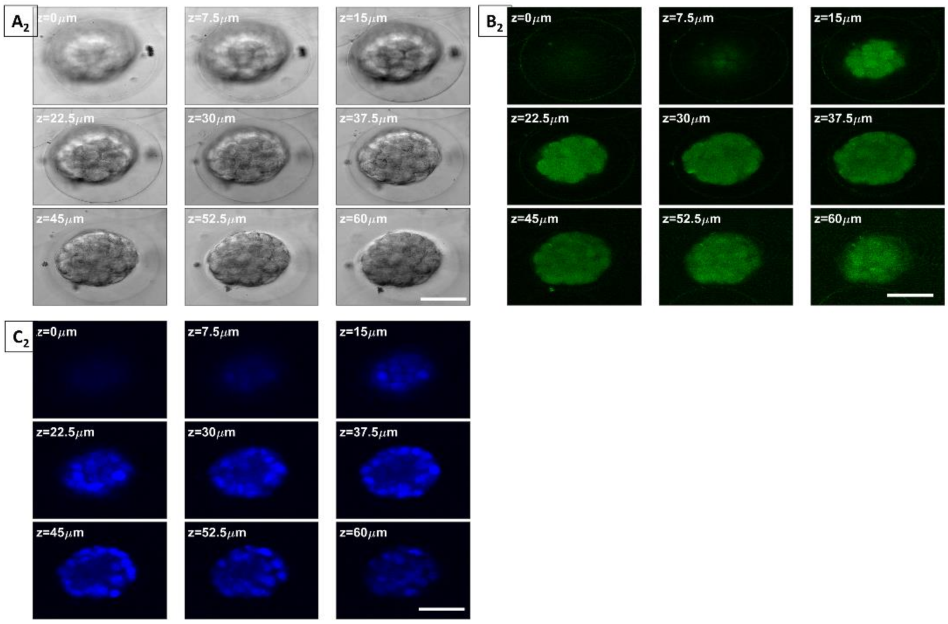

Supplementary Figure S8. Confocal images of HEK293FT spheroids growing in alginate gradient beads (ranging from 0.75% to 3% concentration) at day 8. **A.** Bright-field, **B.** Fluorescein diacetate stain, **C.** Hoechst dye stain. Scalebars: 50 µm.

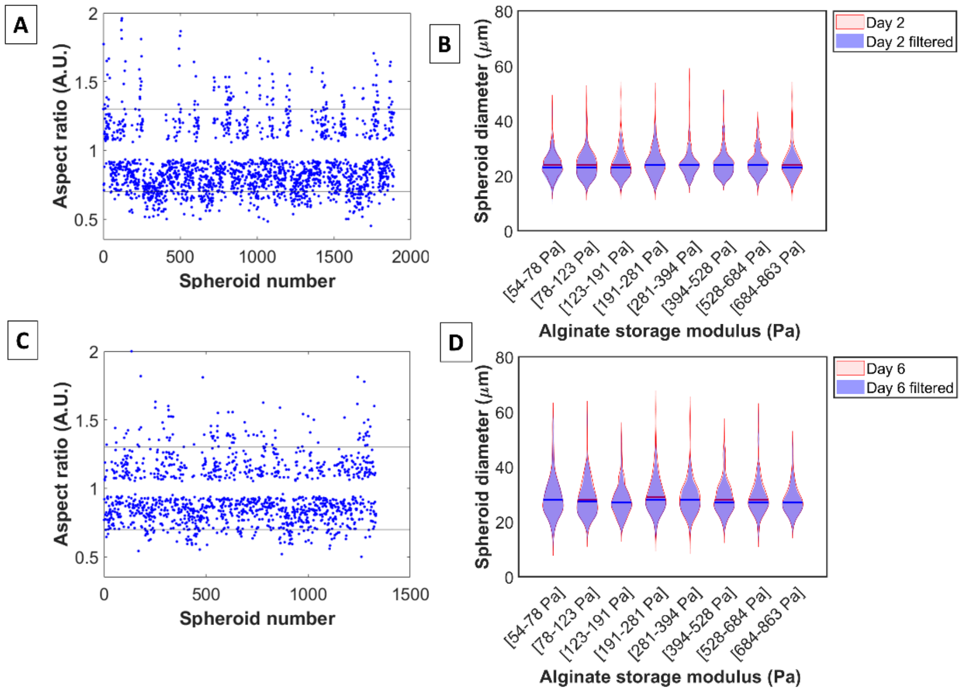

Supplementary Figure S9. A & C: Aspect ratio of YOLOv4 spheroid detections (length of box/width of box) for spheroids at days 2 and 6. The grey lines represent the thresholds (0.7-1.3) for bead selection for further analysis. **B & D:** Violin plot comparison of estimated spheroid diameter versus alginate stiffness at days 2 and 6 when excluding spheroids with detection aspect ratio above 1.3 and below 0.7 (labelled 'filtered' in the legends).

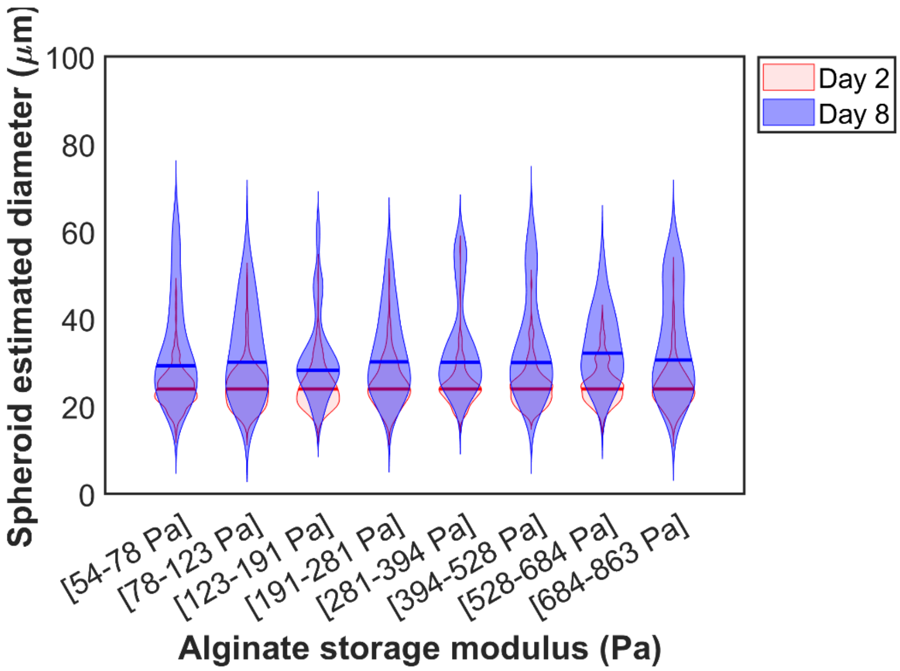

Supplementary Figure S10. Violin plots for the projected spheroid area against the stiffness of alginate at day 2 and day 8. The medians of the distributions are represented by horizontal lines of the corresponding color.

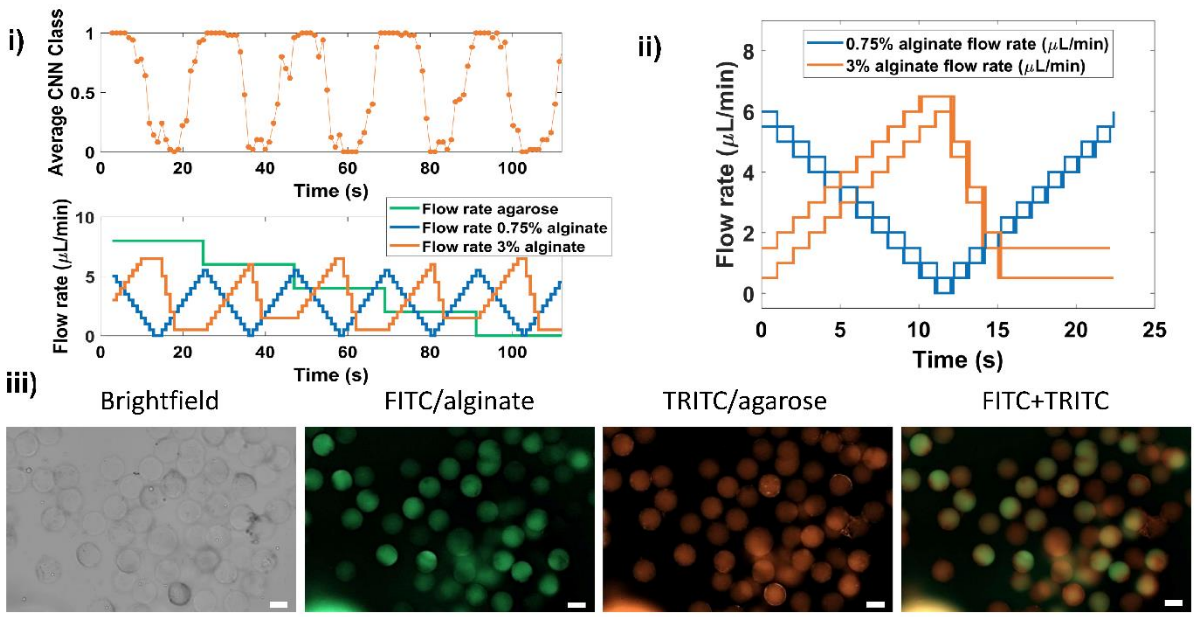

Supplementary Figure S11. 2-dimensional gradient of alginate/agarose hydrogel beads. **i)** An example of 5 consecutive gradients showing the measured CNN classes and corresponding (figure below) evolution of flow rates for the 1% agarose solution and low and high viscosity alginate solutions. **ii)** Overlay of flow rate profile for 5 consecutive gradients. **iii**) The presence of alginate is indicated by the green fluorescence channel. The red fluorescent channel represents agarose stained with TRITC dye. Scale bar: 50 µm.

Supplementary Movie

Supplementary Movie S1 shows the intersection between the two alginate streams containing cells during gradient formation.
